# Future Temperature Extremes Will Be More Harmful: A New Critical Factor for Improved Forecasts

**DOI:** 10.3390/ijerph16204015

**Published:** 2019-10-20

**Authors:** Varotsos Costas A, Mazei Yuri A

**Affiliations:** 1 Climate Research Group, Division of Environmental Physics and Meteorology, Faculty of Physics, National and Kapodistrian University of Athens, University Campus Bldg. Phys. V, 15784 Athens, Greece; 2 Department of General Ecology and Hydrobiology, Lomonosov Moscow State University, Leninskiye gory, 1, 199991 Moscow, Russia; yurimazei@mail.ru

**Keywords:** extreme temperature events, biological indicators, climate change, public health

## Abstract

There is increasing evidence that extreme weather events such as frequent and intense cold spells and heat waves cause unprecedented deaths and diseases in both developed and developing countries. Thus, they require extensive and immediate research to limit the risks involved. Average temperatures in Europe in June–July 2019 were the hottest ever measured and attributed to climate change. The problem, however, of a thorough study of natural climate change is the lack of experimental data from the long past, where anthropogenic activity was then very limited. Today, this problem can be successfully resolved using, inter alia, biological indicators that have provided reliable environmental information for thousands of years in the past. The present study used high-resolution quantitative reconstruction data derived from biological records of Lake Silvaplana sediments covering the period 1181–1945. The purpose of this study was to determine whether a slight temperature change in the past could trigger current or future intense temperature change or changes. Modern analytical tools were used for this purpose, which eventually showed that temperature fluctuations were persistent. That is, they exhibit long memory with scaling behavior, which means that an increase (decrease) in temperature in the past was always followed by another increase (decrease) in the future with multiple amplitudes. Therefore, the increase in the frequency, intensity, and duration of extreme temperature events due to climate change will be more pronounced than expected. This will affect human well-being and mortality more than that estimated in today’s modeling scenarios. The scaling property detected here can be used for more accurate monthly to decadal forecasting of extreme temperature events. Thus, it is possible to develop improved early warning systems that will reduce the public health risk at local, national, and international levels.

## 1. Introduction

Several disciplines such as biology, ecology, and epidemiology co-operate to maintain environmental quality and public health at desirable levels of safe living. In some cases, several indicators of the past environment indicate the current and future environmental state. To exploit this fact, in-field research is currently being conducted in order to identify such indicators with sufficient reliability. An example of such research focuses on chironomids (the name comes from the ancient Greek word *kheironómos*), which includes a worldwide distributed family of nematoceran flies. This is a large taxon of mosquito-like insects, which is important as an indicator of water properties. For instance, chironomids can indicate whether pollutants are present in a lake. Due to their environmental sensitivity, their fossils are widely used by paleolimnologists as indicators of past climatic variability [1,2,3,4,5].

Climate reconstruction over long timescales is crucial in investigating the processes that affect the climate system. In this context, the chironomids, kept in lake sediments, allow for the quantitative production of summer air temperature reconstructions with high resolution and low error. Analysis of modern surface sediments from one hundred lakes of high-latitude in northern European Russia to central Siberia has shown that chironomid distribution was primarily driven by July air temperatures [6]. This sensitivity enabled the development of a chironomid-inference model for the reconstruction of air temperature in July. This is in close agreement with the local instrumental records, suggesting that models can reliably reproduce the past climate [7,8,9].

Therefore, the analysis of biological records can shed light on the properties of past climate variability. At that time, there were no experimental measurements and the environmental impact of anthropogenic activity was not significant. Recent studies have shown that the Arctic has warmed faster than any other region in the last century. This warming causes a series of biotic and abiotic changes that directly affect ecosystems [10,11,12]. For example, large fluctuations in temperature in successive days affect environmental quality (e.g., air pollution) and public health. This has been widely recognized by environmental and epidemiological researchers for environmental and health risk assessment [2,3,12].

The air temperature spontaneously creates fluctuations with high variability in frequency, amplitude, duration, and recurrence. However, little is known about whether air temperature fluctuations reflect a long memory of the climate system. In particular, it is very interesting to investigate whether a slight temperature disturbance at some time in the past may cause a later disturbance with magnified amplitude in accordance with the power-law (i.e., exhibiting the scaling effect). If this were the case, the overlap of anthropogenic activity with the natural temporal variability of air temperature would therefore generate an enhanced response later on.

It should therefore be of interest to investigate the specific features of the temporal dynamics of summer air temperature, when heatwave events often occur. This has to be done by applying modern methods of statistical physics, as the climate is considered a complicated (with many degrees of freedom) and complex (non-linear, dynamical, sensitive) system (e.g., [13,14,15,16]).

Following the afore-mentioned hypothesis, Varotsos et al. [17] demonstrated a compelling example of the scaling effect on a sea surface temperature time-series during 1948–2012, and the Niño3.4 index for the period 1948–2012. Scaling behavior was also found in the marine interstitial ciliate community when analyzing the characteristics of the marine interstitial ciliate community in the White Sea intertidal sand during 1991–2011 [18].

The purpose of the present paper was to investigate the temporal correlations of fluctuations in summertime air temperature at time scales ranging from one to several years. Our analyses focused on whether these fluctuations obey power-law scaling behavior. More specifically, we investigated the existence of scaling property in the temporal variability of summer temperature for the period 1181–1945. This was based on a high-resolution quantitative reconstruction derived from biological records of sediments of Lake Silvaplana (Switzerland). The results will indicate the presence or absence of the scaling effect on the air temperature at a time when the anthropogenic impact on the environment was not as large as it is today. In the case of the presence of scaling effect, it will be attributed to natural factors and not to human activities. The inclusion of this scaling property in the forecasting algorithms of extreme weather effects modeling (e.g., heatwaves/coldwaves) will increase the reliability of early warning systems. Additionally, this will help policy makers to improve the national and regional addressing of pertinent issues synergistically affecting environmental and human health (e.g., high temperatures can lead to asthma attacks).

## 2. Material and Methods

For the needs of the present paper described above, we used summer temperature (June–August) for the period 1181–1945, based on a high-resolution quantitative reconstruction derived from the biological records of the lake’s Silvaplana sediments [19]. More precisely, the reconstruction resulted from the combination of the annually analyzed biogenic silica data and decadal chironomid-inferred temperatures from the sediments of the proglacial Lake Silvaplana (south-eastern Swiss Alps, coordinates: 46°27’N, 9°48’E, altitude: 1800 m). The sediment of Lake Silvaplana is laminated annually and is considered a good predictor of summer temperatures in large parts of western and central Europe. A detailed description of the material and methods used for this reconstruction is given by Trachsel et al. [20].

**Figure 1:**
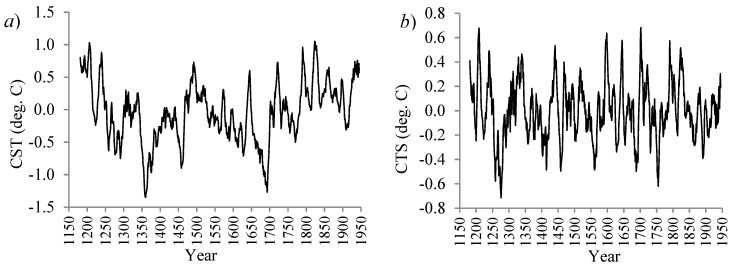
(**a**) The 765-years combined summer temperature (CST) record and (**b**) the deseasonalized and detrended CST time-series during the period 1181–1945, obtained from the sediments of the proglacial Lake Silvaplana (south-eastern Swiss Alps, coordinates: 46°27’N, 9°48’E, altitude: 1800 m).

The record of combined summer temperature (CST) during the period 1181–1945 is illustrated in Figure 1a and shows strong (multi) decadal-scale variability overlapped with long-term trends and seasonal cycles. Linear least squares regression and the Wiener filter were used to eliminate trend and seasonality, respectively [21] (see Figure 1b).

To investigate whether the CST time-series exhibited long-term power-law and multifractal behavior, we used detrended fluctuation analysis (DFA) and multifractal detrended fluctuation analysis (MF-DFA) [22,23,24,25,26,27,28,29,30,31,32,33].

The key operations of both MF-DFA and DFA tools are briefly described below.

The first operation focuses on the integration of the initial time-series. This is accomplished by calculating the deviations of the observations from their mean value. The integrated time-series is then sub-divided into non-overlapping boxes of equal length.

The second operation is to calculate the best polynomial fit with the corresponding variance in each box:

(1) $$\mathrm{for}\;\mathrm{each}\;\mathrm{box}\;k=1,\;\ldots ,\;N_{s}:\;F^{2}(k,s)=\frac{1}{s}\sum_{i=1}^{s}{\left[x((k-1)s+i)-\ell (i)\right]}^{2}$$

where *x*(*i*) is the integrated time-series; ℓ(i) is the polynomial (of order *l*) least-square fit to the *s* data; and *N* is the observations.

The same is repeated, starting at the end of the time-series:

(2) $$\mathrm{for}\;\mathrm{each}\;\mathrm{box}\;k=N_{s}+1,\;\ldots ,\;2N_{s}:\;F^{2}(k,s)=\frac{1}{s}\sum_{i=1}^{s}{\left[x((N-k-N_{s})s+i)-\ell (i)\right]}^{2}$$

The third operation is to calculate the fluctuation function by averaging the variances in all boxes:

(3) $$F_{q}(s)={\left[\frac{1}{2N_{s}}\sum_{k=1}^{2N_{s}}{\left[F^{2}(k,s)\right]}^{q/2}\right]}^{1/q}\mathrm{and}\;F_{o}(s)=\exp\left[\frac{1}{2N_{s}}\sum_{k=1}^{2N_{s}}In[F^{2}(k,s)]\right],\;\mathrm{when}\;q\to 0$$

In Equation (3), *q* denotes the variable moment, which becomes negative (positive) for small (large) fluctuations, respectively.

The last operation is to plot log*F_q_*(*s*) against log *s* for various *q* values in order to derive a power-law behavior for *F_q_*(*s*), i.e.,:

(4) $$F_{q}(s)\sim s^{h(q)}$$

where *h*(*q*) is the slope of the regression line (i.e.,: the generalized Hurst exponent).

It should be emphasized that MF-DFA is a generalization of the detrended fluctuation analysis (DFA) resulting from Equation (3) for *q = 2*). The single fractal DFA-exponent *a(≡h(2))* identifies homogeneous fractal characteristics over all time scales [26,27].

In the case of monofractal dynamics, all *h(q)* scatter around *α* and *F_d_* (*s*) for *q = 2* obey a power-law: *F_d_* (*s*) ~ *s^a^*. Specifically, an exponent $\frac{1}{2}$ < *α* < $\frac{3}{2}$ is a signal of the persistent power-law correlations. Finally, the findings *α* = 1 and *α* = $\frac{3}{2}$ depict the 1/f noise and the Brownian motion, respectively.

It is important to note that the straight line fit to the fluctuation function does not confirm the scaling effect existence. It is necessary to study the power spectral density and the stability of the “local slopes” *a*(*s*) by applying a straight line fit on log*F_d_*(*s*) versus log *s* in a small window shifting across all *s* scales [13]. To evaluate the stability of “local slopes”, we applied the method used by Varotsos et al. [17].

## 3. Results and Discussion

### 3.1. The Long Memory Effect in Ambient Temperature

**Figure 2:**
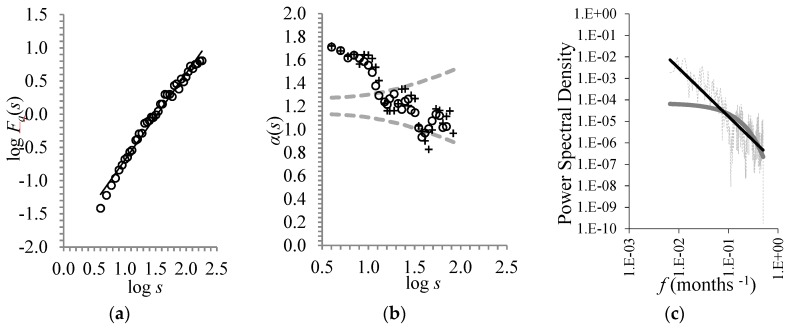
(**a**) The root-mean-square fluctuation function *F_d_*(*s*) of the CST time-series as a function of time scale *s* (in years). The corresponding best fit equation is *y* = 1.30x − 1.99 (*R*² = 0.98). (**b**) The variability of the power-law exponent expressed as local slopes of the log*F_d_*(*s*) vs. log *s* calculated within a window of 12 points (circles) and 10 points (crosses). The dashed grey line represents the 2*s_a_* intervals around the local slopes with an average value of $\bar{a}$ = 1.2. (**c**) The power spectral density as a function of frequency (a) (dashed gray line) with a power-law fit (black line) (*y* = 1·10^−7^·x^−2.24^ with *R*² = 0.56) and the exponential fit (grey line) (*y* = 7·10^−5^·e^−11.5x^ with *R*² = 0.39).

As above-mentioned, the study of the scaling properties of summer temperature is the main purpose of the present paper. To this end, the first part of our analysis was to apply the DFA-method to the CST time-series in order to illustrate the root-mean-square fluctuation function *F_d_*(*s*) against the time scale *s* (see Figure 2a).

The results showed that the DFA-exponent was *a* = 1.3 ± 0.03. This depicts persistent long-range correlations (i.e., the CST fluctuations are correlated at different times) with a magnitude obeying a power-law.

To establish the power-law long-term persistence above-mentioned, we checked the validation of the following two criteria proposed by Maraun et al. [34].

(i) The stability of the exponent of the power-law found above: In this respect, the calculated local slopes *a*(*s*) vs. log *s* (depicted in Figure 2b, for two different 10- and 12-point window sizes) are in the range $\left(\bar{a}-1.96s_{a},\;\bar{a}+1.96s_{a}\right)$ (where $\bar{a}$ is the average value and *s_a_* is the standard deviation of the local slopes *a*(*s*)). This demonstrates the stability of the power-law exponent to a sufficient range (i.e., after 41 years).
(ii) The hyperbolic best fit of the power spectral density as a function of frequency: The best fit of the profile of the power spectral density vs. frequency is a hyperbolic curve rather than an exponential one (Figure 2c).

Consequently, both criteria of Maraun et al. [34] were satisfied by the CST time-series. Therefore, we found that, in fact, average temperature fluctuations exhibit a power-law behavior, or in other words, the temperature is characterized by a long memory that reaches almost four decades.

**Figure 3:**
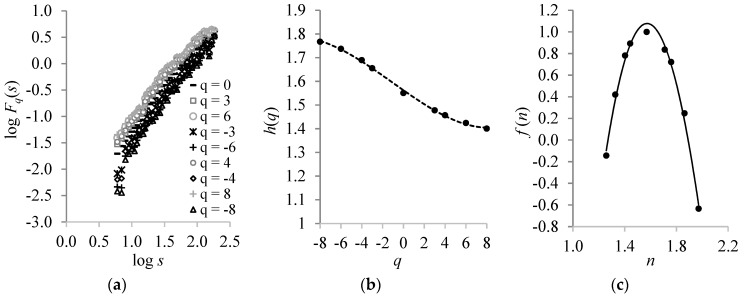
(**a**) The multifractal detrended fluctuation analysis (MF-DFA) fluctuation factor *F_q_*(*s*) of the CST time-series as a function of the time scale *s* of particular moments *q*. (**b**) The variability of the generalized Hurst exponent *h*(*q*) with respect to *q*-values. The best fit was achieved by the polynomial *h(q)* = 1·10^−4^*q*^3^ + 0.0004*q*^2^ − 0.03*q* + 1.56, with *R*² = 1.00. (**c**) The variability of the singularity spectrum *f*(*n*) versus singularity strength *n*, with a best fit expressed by the equation: *f(n) =* 1.74*n*^3^ − 19.48*n*^2^ *+* 48.35*n −* 33.57, with *R*² *=* 0.99.

Furthermore, it was of interest to investigate the memory effect for low and high temperature fluctuations. For this reason, we applied the MF-DFA technique to the CST time-series. The findings were as follows:

(i) A power-law behavior was yielded by the *q*-th order fluctuation function *F_q_*(*s*). The slopes of the linear regressions of log*F_q_*(*s*) vs. log *s* were found to be higher for the negative moments than for the positive ones, thus revealing multi-scaling dynamics (Figure 3a).
(ii) The generalized Hurst exponent *h*(*q*) varied with *q* in a manner that *h*(*q*) > 0.5 establishing the power-law long-range correlations in the CST (Figure 3b).
(iii) The fact that the *h*(*q*) values for the negative *q-values* were higher than the positive ones indicate that the CST exhibited multifractality with different behavior for the positive and negative moments (Figure 3b). This means that low and high temperature fluctuations obey the power-law with different exponents.

### 3.2. The Long Memory Effect in the Background of Ambient Temperature

In the previous subsection, we presented the analysis of the summertime temperature during 1181–1945, according to which it exhibited long memory. It would be interesting to repeat the same analysis to the same timeseries, but eliminate any long-term trend and natural cycles. This will help to understand the source of the long-term memory found above. In other words, we are interested in whether this long-memory arises from external forcings (e.g., solar activity, anthropogenic activity, etc.) or from the intrinsic properties of the thermal regime.

**Figure 4:**
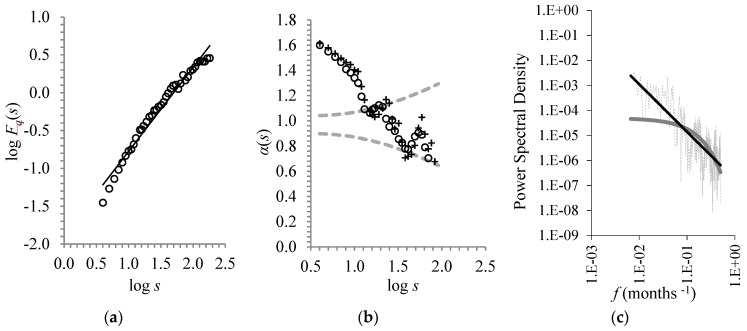
As in Figure 2, but for the detrended and deseasonalized CST time-series. (**a**) The best fit equation was *y* = 1.1x – 1.88 (*R*² = 0.97). (**b**) The mean value of the local slopes was $\bar{a}$ = 1. (**c**) The power-law fit (black line) was *y* = 2·10^−7^·x^−1.90^ (*R*² = 0.53) and the exponential fit (grey line) was *y* = 5·10^−5^·e^−9.9x^ (*R*² = 0.39).

With this in mind, we applied the same analysis as described above to the detrended (trend subtracted) and deseasonalized (natural cycles subtracted) CST time-series. The DFA-exponent found *a* = 1.1 ± 0.03 to indicate, as above, persistent power-law long-range correlations (Figure 4a). This time, the DFA exponent was close to *a* =1 of 1/f-noise, which is often observed in many geophysical phenomena.

This long-term persistence was established by testing the two criteria proposed by Maraun et al. [34]. In more detail, the local slopes *a*(*s*) vs. log *s* (shown in Figure 4b, for the 10- and 12-point windows) again appeared to belong to the range $\left(\bar{a}-1.96s_{a},\;\bar{a}+1.96s_{a}\right)$ that showed stability over a sufficient range (after 32 years). Furthermore, the profile of the power spectral density versus frequency was better adapted to the power-law rather than exponential decay (Figure 4c).

**Figure 5:**
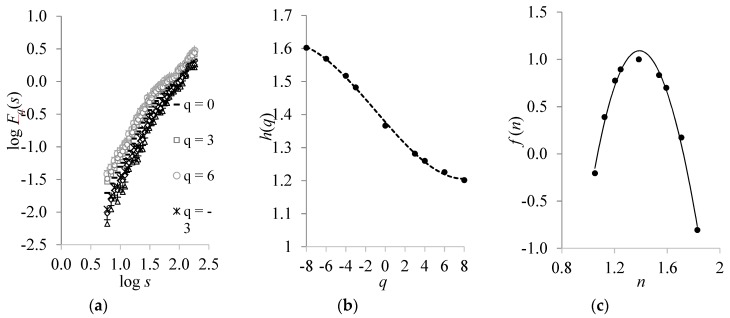
As in Figure 3, but for the detrended and deseasonalized CST time-series. (**a**) In (**b**), the empirical curve (dots) was fitted by the polynomial *h(q)* = 1·10^−4^*q*^3^ + 4·10^−4^*q*^2^ − 0.03*q* + 1.38, with *R*² = 1.00. In (**c**), the empirical curve (dots) was fitted with *f(n) =* 1.61*n*^3^ − 17.06*n*^2^ *+* 38.06*n −* 23.18, with *R*² *=* 0.99.

To investigate the order of magnitude of the power-law exponent for low and high temperature fluctuations, we applied the MF-DFA technique to the detrended and deseasonalized CST time-series. The findings of Figure 5a–c were similar to those of Figure 3a–c, again indicating multifractality and power-law long-range correlations.

The result of the analysis presented in this subsection is that the long-memory effect in summertime temperature stems from the intrinsic properties of the thermal regime and not from its trend or natural cycles. In other words, long-term memory at ambient temperature is independent of natural or external influences. Consequently, the expected impacts on ecosystems and public health from extreme temperature events depend on the temperature itself and will increase their intensity according to the above-mentioned power law.

### 3.3. The Exploitation of the Scaling Effect to Develop Improved Early Warning Systems for Air Temperature Extremes

Forecast models and early warning systems of air temperature extremes contain climatological algorithms that are an important part of their fidelity. The air temperature scaling effect described in detail in the above analysis is a key feature that optimally revises the climatologies currently used to reflect all regular/irregular and nonlinear/linear components.

The resulting innovative power-law equations can be incorporated into the climatological algorithms of the operational early warning systems. This has to be done to adjust the existing bias between the direct forecast models output and the actual observed heatwave/coldwave parameters on a global scale. Strictly assessing this bias, improved early warning systems could give a potential bias correction to achieve more reliable, in advance alerts that can be provided to users and decision-makers.

The afore-mentioned power-law equations can also contribute substantially to the development of improved nowcasting of the extreme temperature events. By nowcasting extreme temperature events, we refer to the use of air temperature observations to estimate the current dynamic state of the temperature regime. In other words, a nowcasting extreme event differs from forecasting as it aims to assess the current state of the temperature regime and not the probability of a future extreme event. We are currently working to develop a novel nowcasting model for extreme air temperature events that will be based on the scaling effect presented in this paper and the new methods already developed by our research team [35,36,37,38]. This innovative nowcasting tool will be described in an upcoming paper.

## 4. Conclusions

High temperature events are often mixed with other meteorological and air-pollution conditions causing heat-related deaths [39]. These excessive heat events occur in the summertime period. For this reason, the analysis described above was carried out by only considering the summertime data.

To examine whether the summertime regime was characterized by long memory (i.e., the cause and effect occur at different times with the latter being magnified by the power-law), the reconstructed data of the summer temperatures for the period 1181–1945 were analyzed. The main results obtained from this analysis are as follows:

(i) The temperature record revealed persistent a power-law behavior with a single exponent over all time scales. This means that an average fluctuation in temperature in the past may create another future temperature fluctuation after several decades, with enhanced amplitude that can be calculated from the resulting power-law.
(ii) Small and large fluctuations in temperature obey the power laws with different exponents.

The detrended and deseasonalized temperature time-series (trend and natural cycles removed) exhibited a 1/*f*-type power-law over all time scales (e.g., as in the case (i) above). It is worth noting that the latter was found in several geophysical parameters.

In summary, the investigated temperature fluctuations exhibited persistent long-range correlations. This result shows that the elimination of trend and known natural cycles from the temperature time series during the period 1181–1945 simply provides the so-called “noise”. The “noise” must not be ignored, but with proper analysis can provide a very important message. Our results show that analyzing the temperature “noise” with the tools above-mentioned reveals the following: a slight temperature change in the past has a magnified effect on the current or future temperature regime within a 30–40-year time window. The order of magnitude of this magnification is given by the power-laws derived above. It is also important to note that the same message can be received without removing the trend and natural cycles if the analysis described above is applied.

Consequently, temperature fluctuations after 1945 will be a combined result of the anthropogenic activities (reinforced after 1945), and the existing background of the fluctuations (“noise”). In other words, the expected impact of climate change on the temperature increase in the future will not only depend on current anthropogenic activities. There is a background to temperature variability before 1945 that will respond late to the causes that occurred in the past. Therefore, the risk of heat-related diseases in the future is much higher than we would expect with current models [40]. These results need to be considered when developing new advanced forecasting models and early warning systems in order to increase their fidelity to adaptation techniques and policies to protect public health. Indeed, it is necessary to evaluate the quality of the climatology used as a reference to a forecasting model and an early warning system, before assessing the reliability of the predictability of events. In particular, the afore-mentioned scaling effect and its calculated metrics must be taken into account when comparing temporal climatologies in order to develop new forecasting models and improved early warning systems.
