# Erratum: Future Temperature Extremes Will Be More Harmful: A New Critical Factor for Improved Forecasts

**DOI:** 10.3390/ijerph17093288

**Published:** 2020-05-08

**Authors:** Costas A. Varotsos, Yuri A. Mazei

**Affiliations:** 1 Climate Research Group, Division of Environmental Physics and Meteorology, Faculty of Physics, National and Kapodistrian University of Athens, University Campus Bldg. Phys. V, 15784 Athens, Greece; 2 Department of General Ecology and Hydrobiology, Lomonosov Moscow State University, Leninskiye gory, 1, 199991 Moscow, Russia; yurimazei@mail.ru

The authors would like to correct the names and surnames of both authors of their previous paper [1] as follows:

Costas A. Varotsos ^1,2,^* and Yuri A. Mazei ^2^

Therefore, to cite this paper please use the correct reference as follows:

Varotsos, C.A.; Mazei, Y.A. Future Temperature Extremes Will Be More Harmful: A New Critical Factor for Improved Forecasts. *Int. J. Environ. Res. Public Health* **2019**, *16* (20), 4015.
